# Anion-Exchange Membranes’ Characteristics and Catalysts for Alkaline Anion-Exchange Membrane Fuel Cells

**DOI:** 10.3390/membranes14120246

**Published:** 2024-11-22

**Authors:** Fa-Cheng Su, Hsuan-Hung Yu, Hsiharng Yang

**Affiliations:** 1 Graduate Institute of Precision Engineering, National Chung Hsing University, Taichung City 402, Taiwan; 06250625frank@gmail.com (F.-C.S.); apple6359666@gmail.com (H.-H.Y.); 2 Innovation and Development Center of Sustainable Agriculture (IDCSA), National Chung Hsing University, Taichung City 402, Taiwan

**Keywords:** alkaline anion-exchange membrane fuel cell, anion-exchange membrane, non-platinum group metal, membrane electrode assembly

## Abstract

This work aims at the effects of anion-exchange membranes (AEMs) and ionomer binders on the catalyst electrodes for anion-exchange membrane fuel cells (AEMFCs). In the experiments, four metal catalysts (nano-grade Pt, PtRu, PdNi and Ag), four AEMs (aQAPS-S8, AT-1, X37-50T and X37-50RT) and two alkaline ionomers (aQAPS-S14 and XB-7) were used. They were verified through several technical parameters examination and cell performance comparison for the optimal selection of AMEs. The bimetallic PdNi nanoparticles (PdNi/C) loaded with Vulcan XC-72R carbon black were used as anode electrodes by using the wet impregnation method, and Ag nanoparticles (Ag/C) were used as the catalyst cathode. It was found that the power density and current density of the X37-50RT are higher than the other three membranes. Also, alkaline ionomers of XB-7 had better performance than aQAPS-S14. The efficiency was improved by 32%, 155% and 27%, respectively, when compared to other membranes by using the same catalyst of PdNi/C, Ag/C and Pt/C. The results are consistent with the membrane ion conductivity measurements, which showed that the conductivity of the X37-50RT membrane is the highest among them. The conductivity values for hydroxide ions (OH^−^) and bromide ions (Br^−^) are 131 mS/cm and 91 mS/cm, respectively. These findings suggest that the properties (water uptake, swelling rate and mechanical) of the anion-exchange membrane (AEM) can serve as a key reference for AEM fuel cell applications.

## 1. Introduction

Fuel cell technology is increasingly recognized as an alternative energy source to fossil fuels. Among them, proton-exchange membrane fuel cells (PEMFCs) are suitable for everyday applications due to their relatively low temperature and atmospheric pressure characteristics, as well as their lack of chemical hazards to humans and harm to the environment. They are being developed for use in transport, stationary, and portable systems. However, the need for costly platinum (Pt) as the catalyst layer for both the anode and cathode, along with the acidic nature of the electrolyte, leads to the rapid corrosion of most metals. In contrast, alkaline anion-exchange membrane fuel cells (AEMFCs) possess the advantages of proton-exchange membrane fuel cells and improve material stability in acidic environments due to their alkaline nature. This allows for the use of less expensive non-platinum catalysts and fuel cell components, indicating that AEMFCs represent a trend for future development. In recent years, AEMFCs have attracted the attention of experts in the fuel cell field as a low-cost alternative to PEMFCs [1,2,3]. AEMFCs exhibit rapid oxygen reduction reaction (ORR) kinetics in alkaline medium [4,5,6], making them a potential alternative for using non-platinum catalysts (such as Ag, Pd and Ni) and mitigating potential corrosion issues. However, the commercialization of AEMFCs is still hindered by two main technical challenges: lower electrical conductivity [7] and lower chemical stability of the anion-exchange membranes (AEMs) [8,9]. Therefore, the development of AEMs with high electrical conductivity [10] and good mechanical and chemical stability, along with the use of non-platinum catalysts [11,12], represents a major research challenge for alkaline fuel cells [13,14].

Using AEMs in hydrogen fuel cells can eliminate carbonate issues and avoid liquid electrolyte management [15]. Therefore, countries around the world are committed to developing AEMFCs. Due to their advantages over acidic PEMFCs in terms of cathode kinetics, ohmic polarization, low cost, stability and material durability [16], the AEMFC is considered an alternative to the PEMFC and has gained attention. Currently, many researchers are focusing on the synthesis of polymer alkaline anion-exchange membranes with high ionic conductivity and excellent chemical stability, as well as the development of favorable and low-cost novel electrocatalysts, such as silver (Ag) and nickel (Ni), which are expected to significantly reduce the cost of fuel cell technology [12].

AEMFCs have basic components of an anode, cathode and solid polymer electrolyte, using hydrogen and oxygen as the anode and cathode sides, respectively [17,18]. The anode and cathode are separated by an AEM to prevent hydrogen from penetrating the cathode, which leads to fuel loss and mixed potentials. At the same time, it establishes a pathway for conductive hydroxide ions, while the external circuit provides a channel for electrons to form a loop. The performance depends on three main parameters: The electrocatalysts used for the hydrogen oxidation reaction (HOR) and the ORR, which determine the electrochemical kinetics [19]; the structure of the membrane electrode assembly (MEA) that affects mass, ion and electron transport [19]; the operating conditions that influence electrochemical and transport behavior as well as power output. On the anode, humidified hydrogen gas is supplied to the anode flow channel and transported through the anode gas diffusion layer (GDL), ultimately reaching the anode catalyst layer (CL). Under the catalysis of the catalyst, hydrogen molecules react with hydroxide ions through solid polymer conduction to produce water and electrons. The anode reaction is shown in Equation (1):

2H_2_ + 4OH^−^ → 4H_2_O + 4e^−^  *E*^0^ = −0.83 V (1)

On the cathode, humidified oxygen supplied through the cathode flow channel is delivered to the cathode CL from cathode GDL, where water and oxygen undergo a reduction reaction to produce hydroxide ions as shown in Equation (2):

O_2_ + 2H_2_O + 4e^−^ → 4OH^−^  *E*^0^ = 0.40 V (2)

The overall reaction combining the HOR given by Equation (1) and the ORR given by Equation (3) is represented as follows.

2H_2_ + O_2_ → 2H_2_O  *E*^0^ = 1.23 V (3)

From the above reaction, it can be seen that the final product of the AEMFC is water, which is an environmentally friendly way to generate electricity. In addition, hydrogen and oxygen need to be humidified before entering the battery to achieve a better performance, for the following main reasons: One of the main drawbacks of AEM is that its conductivity is sensitive to relative humidity. Therefore, the humidified gas supplied to the channel can humidify the exchange membrane, enhancing ionic conductivity and reducing impedance. Considering the electrochemical reactions at the cathode, humidified oxygen forms hydroxide ions, which require water molecules.

Carbon black provides excellent electrical conductivity performance, offering high conductivity at relatively low load levels [18]. It has good processability, a low sulfur content and low ionic contamination. Carbon black consists of many carbon black particles that aggregate into chain-like or grape-like structures. The structure is determined by the size, shape and number of particles in each aggregate, and carbon black composed of aggregates is referred to as high-structure carbon black [20]. The oil absorption value is commonly used to indicate the structure; the higher the oil absorption value, the higher the structural integrity of the carbon black, making it easier to form spatial network channels that are not easily destroyed. High-structure carbon black particles are fine, have a large surface area and are densely packed, which is beneficial for forming conductive structures within polymers. Pt is a shiny, ductile silver-white metal, and its primary use is as a catalyst in chemical reactions, typically in the form of Pt/C. It has the highest ductility among all pure metals, surpassing gold, silver and copper, but its malleability is lower than that of gold. Platinum metal has excellent corrosion resistance and is very stable at high temperatures, with stable electrical properties as well. Therefore, the metal catalysts used in fuel cells predominantly use platinum [21], which is currently the most effective metal. However, due to its high price as a precious metal, researchers are seeking alternative catalysts. Ag exhibits high ORR activity at a high pH and has greater stability and cost-effectiveness within a temperature range [22,23]. It was found that in high-concentration alkaline media, the ORR activity of silver is close to that of platinum [24]. It was successfully prepared with silver molybdate electrocatalysts using a hydrothermal method [25]. Compared to commercial Pt/C catalysts in alkaline solutions, the synthesized silver molybdate electrocatalysts exhibit higher ORR activity and durability. Additionally, the price of silver is much lower than that of the precious metal platinum (about 75 times cheaper). These advantages make silver a potential candidate for applications in alkaline fuel cells and metal–air batteries. Blizanac et al. studied the ORR on silver single crystal surfaces in alkaline solutions within the temperature range of 293–333 K using rotating ring-disk technology [4], and their results indicate that the ORR proceeds via four electron transfers, with a very low amount of H_2_O_2_ produced.

Palladium (Pd), as a pure metal, can serve as an alternative to Pt anode catalysts in AEMFCs. The face-centered cubic (FCC) structure of Pd has significant characteristics, such as the same atomic size and electronic configuration as Pt. Pd nanoalloys provide an excellent system to explore the comprehensive effects of alloying and nanoscale size on hydrogen storage, as well as the thermodynamic properties of these materials. In 2014, the synergistic effect of Pd alloy bimetallic catalysts (PdRu and PdIr) or Pd-supported tungsten oxide was reported to enhance HOR activity [26]. However, the stability of Pd under acidic and high-temperature conditions is lower than that of Pt, and the ORR activity of Pd is not as good as that of Pt. It is suggested that other transition elements such as Cu, Fe, Co and Ni can be used to create bimetallic catalysts, which can significantly impact the increase in activity. Combining palladium with abundant transition metals can further enhance power output and reduce battery costs. Nickel (Ni) belongs to a type of non-precious metal and is non-toxic. The electrode materials composed of nickel and palladium show good performance in fuel cells. Density functional theory (DFT) calculations of bimetallic alloys indicate that Pd-doped Ni can weaken the Pd-O bond [26]. Pd and Ni are in the same column of the periodic table, and the solid solutions formed by these elements are similar to the composition of the FCC crystal structure. This high activity on the surface and in the dispersion system of Pd/Ni is crucial for developing HOR electrocatalysts in alkaline media.

The use of palladium–nickel (PdNi) dual-function bimetallic electrocatalysts brings several advantages, such as promoting the desorption of various molecular species and providing higher surface site availability [27]. It has been well established that Pd/Ni alloys exhibit high chemical stability and are more cost-effective compared to other alloys. At the interface between nickel and the electrolyte, nickel nanoparticles provide adsorbed hydroxyl species (OH_ads_) for surface bonding, which is due to the oxygen affinity of the nickel surface under a given pH and anodic potential conditions. The palladium surface is active in the dissociative chemisorption of hydrogen and the bonding of surface hydrogen species (H_ads_). Without the OH_ads_ surface species provided by the nickel nanoparticles, hydrogen atoms adsorbed on palladium cannot be oxidized at a high rate when in contact with alkaline electrolytes, thereby reducing the overall activity of the HOR catalyst that consists solely of palladium. The catalytic performance of bimetallic platinum–ruthenium (PtRu) dual-function catalysts is generally higher than that of monometallic catalysts. Previous studies have shown that the addition of a second metal can provide more sites for the adsorption of oxygen-containing species, facilitating the removal of CO_ads_ at a lower potential and significantly weakening the adsorption of intermediates on the Pt surface [28,29]. Among various bimetallic catalysts, PtRu is considered the most promising bimetallic catalyst for fuel cells [30], as it maximizes the utilization of Pt, alters the electronic structure and chemical properties of metal atoms, and enhances its electrocatalytic performance for the methanol oxidation reaction (MOR) [30,31,32,33,34,35].

Anion-exchange membranes (AEM) form ion pairs with hydroxide ions after being immersed in strong bases (KOH or NaOH). The conduction of hydroxide ions within the membrane relies on moisture and is facilitated by the Grotthuss mechanism, which means that the membrane must contain water [35]. Therefore, the operating temperature of the AEMFC is kept below 80 °C. If the operating temperature is too high, a significant quantity of water will be lost and lead to a decrease in anion conductivity. Compared to PEMs, AEMs use hydroxide ions as the conductive species, so they must operate in a high pH environment. In alkaline media, the kinetics of the oxygen reduction reaction are better, allowing the use of non-precious metals such as palladium, nickel, silver and cobalt as catalysts [36], which reduces catalyst costs. The ion exchange capacity (IEC) is an important parameter in anion-exchange membranes [37]. The IEC is defined as the number of cationic groups contained in a one-unit weight of dry membrane, typically expressed in millimoles (mmol/g) or milliequivalents (meq/g) per gram. The IEC is generally determined by back-titration to infer the number of cations on the polymer. The IEC value is closely related to the membrane’s moisture content, conductivity and swelling rate [38].

This study investigates the effects of different AEMs and catalysts on the AEMFCs. Ag/C, Pt/C, PdNi/C and PtRu/C were used as metal catalysts on electrodes, applied to the GDLs of AEMFCs. The GDLs were coated using different ionic polymers and metal catalysts to explore the impact of the GDL on fuel cell performance under various mixtures, aiming to identify the optimal production parameters for gas diffusion electrodes (GDEs). The physical properties of the anion-exchange membrane are also measured and analyzed. Experimental methods include tests for conductivity, water absorption, swelling rate, mechanical properties and current–voltage polarization performance curve analysis, providing an in-depth analysis of the relationship between the AEMs and the ratio parameters of ionic compounds. This research can establish a comparative dataset linking the characteristics of the anion-exchange membrane with the variations in gas diffusion electrode parameters, which can provide insights for future research and development of AEMFCs.

## 2. Materials and Methods

### 2.1. Materials

The 40 wt% of Pt/C was bought from Johnson Matthey, Taipei, Taiwan. The 40 wt% of 40% Wt Platinum Ruthenium on Vulcan SC-72R (PtRu/C) was bought from the Fuel Cell Store, Bryan, TX, USA. Carbon black (XC-72R) was purchased from Cabot Corp., Boston, MA, USA. The gas diffusion layer containing MPL (GDL-340) was purchased from CeTech, Taichung, Taiwan. Palladium chloride (PdCl_2_), hexahydrate nickel chloride (6H_2_O·NiCl_2_) and potassium hydroxide (KOH) were bought from Uni-Onward Corp., Taipei, Taiwan. Sodium borohydride (NaBH_4_), nitric acid (HNO_3_), silver nitrate (AgNO_3_) and sodium citrate (Na_3_C_6_H_5_O_7_) were bought from Shen Chiu Enterprise Corp., Ltd., Taichung, Taiwan. Isopropyl alcohol (IPA) was bought from Tonghwa Chemical, Taipei, Taiwan. Anion-exchange membranes (Sustainion^®^ X37-50 Grade RT and Sustainion^®^ X37-50 Grade T) and ionomer (Sustainion^®^ XB-7 Alkaline Ionomer 5% in ethanol) were purchased from Dioxide Materials, Boca Raton, FL, USA. Anion-exchange membranes (AT-1 and aQAPS-S_8_) and ionomer (aQAPS-S_14_) were bought from professor Lin Zhuang’s laboratory, College of Chemistry and Sciences, Wuhan University, China. Hydrogen (H_2_) and oxygen (O_2_) were bought from Air Products San Fu Co., Ltd., Taipei, Taiwan.

### 2.2. Methods

#### 2.2.1. Carbon Black Surface Modification

In the recirculation system, 20% nitric acid (HNO_3_) was used to modify the surface of the carbon black Vulcan XC-72R particles at 110 °C for 2 h, generating functional groups on the surface. Next, the carbon black was filtered and washed with deionized water (DI water) until the pH value was neutral to remove any residual nitric acid. Finally, the collected carbon black was dried in an oven at 80 °C for 12 h.

#### 2.2.2. Preparation of 40 wt% Ag/C Cathode Catalyst

Through the chemical reduction method, Ag nanoparticles were prepared using 10 mM silver nitrate solution and 7.4 mM sodium borohydride (reducing agent), with 50 mM sodium citrate serving as a stabilizer to prevent the agglomeration of the silver nanoparticles. Add 200 mg of modified carbon black, then stir for 12 h and filter through the suction filtration. Then, use DI water to wash until the pH value is neutral. The silver nanoparticles reduced from the solution bonded with the oxygen-containing functional groups on the surface of the modified carbon black.

#### 2.2.3. Preparation of 40 wt% PdNi/C Anode Catalyst

To prepare a 50:50 ratio of palladium (Pd) and nickel (Ni), mix the 0.01 M palladium chloride solution with the 0.01 nickel chloride solution in hexahydrate and then add the 200 mg of modified carbon black. Add 0.1 M of sodium hydroxide to adjust the pH value (pH = 10~12), and add sodium borohydride (reducing agents) of 2 mg/mL. Then, stir for 12 h and filter through suction filtration. The nanostructured palladium–nickel bimetallic catalyst was achieved, resulting in a 40 wt.% PdNi/C anode catalyst.

#### 2.2.4. Production of Catalyst Slurry

**Figure 1:**
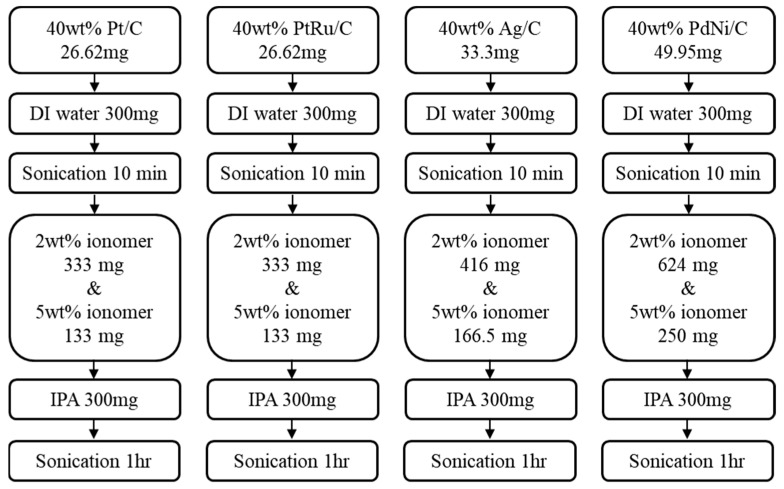
Production procedure of Pt/C, PdNi/C, Ag/C and PtRu/C catalyst slurries.

The GDLs with 3.65 × 3.65 cm^2^ were used in this experiment. The loading of Pt on the anode GDL was 0.8 mg/cm^2^; the loading of PdNi on the anode GDL was 1.5 mg/cm^2^; the PtRu loading on the anode GDL was 0.8 mg/cm^2^; and the Ag loading on the cathode GDL was 1.0 mg/cm^2^. The catalysts used to produce the catalyst slurry included Pt/C, PdNi/C, Ag/C and PtRu/C. The ionomer solutions used were aQAPS-S14 and XB-7, along with deionized water (DI water) and isopropanol (IPA). The function of the ionomer solution is to serve as an ion transport channel between the catalyst layer and the AEM, while IPA acts as a dispersant to ensure the uniform dispersion of the catalyst powder in DI water, as shown in Figure 1.

#### 2.2.5. Production of GDEs

The catalyst slurry was coated on the surface area of 3.65 × 3.65 cm^2^ GDL-340. It was coated separately with the Pt loading of 0.8 mg/cm^2^, the PtRu loading of 0.8 mg/cm^2^, the Ag loading of 1 mg/cm^2^, and the PdNi loading of 1.5 mg/cm^2^. After coating the slurry on GDL-340 on an 80 °C hot plate to dry them, we immersed them individually in a 1 M KOH solution for 24 h; then, the GDEs were finished.

#### 2.2.6. AEMs Attached Hydroxide Ion (OH^−^)

Four different anion-exchange membranes (aQAPS-S8, AT-1, X37-50RT and X37-50T) were used in the experiments. Each membrane was cut into 4 × 4 cm^2^ pieces and treated according to the activation conditions for each membrane to convert the AEM-attached hydroxide ion (OH^−^). The aQAPS-S8 was soaked in a 1 M KOH solution at room temperature for 24 h. The AT-1 was soaked in a 1 M KOH solution at 60 °C for 48 h, which was replaced with the 1 M KOH solution every 24 h. The X37-50RT was soaked in a 1 M KOH solution at room temperature for 48 h, with the solution replaced every 24 h. The X37-50T was soaked in a 1 M KOH solution at room temperature for 10 h.

### 2.3. Material Characterizations

The water uptake, swelling rate, and conductivity of AEMs must be measured after replacing the anions in the membrane with OH^−^. The ion exchange step involves soaking the membrane in a 1 M KOH solution for the required number of days, with a new KOH solution being replaced every 24 h. Finally, the membrane is soaked in ultra-pure water. This experiment used a potentiostat from the National Taiwan University of Science and Technology and a programmable constant temperature and humidity chamber for measurements (MHK), and the membranes were treated by soaking them in OH and BR solutions, respectively. The XRD pattern was measured by a BRUKER D8 DISCOVER SSS from 20 degrees to 90 degrees. Electrochemical characterizations were examined from CHI 600 E. The SEM images were taken by JSM-6700F. AEMFC tests were measured by the fuel cell test station (FCED-PD5).

## 3. Results and Discussion

### 3.1. Catalyst Analysis

#### 3.1.1. X-Ray Diffraction Pattern of PdNi/C

**Figure 2:**
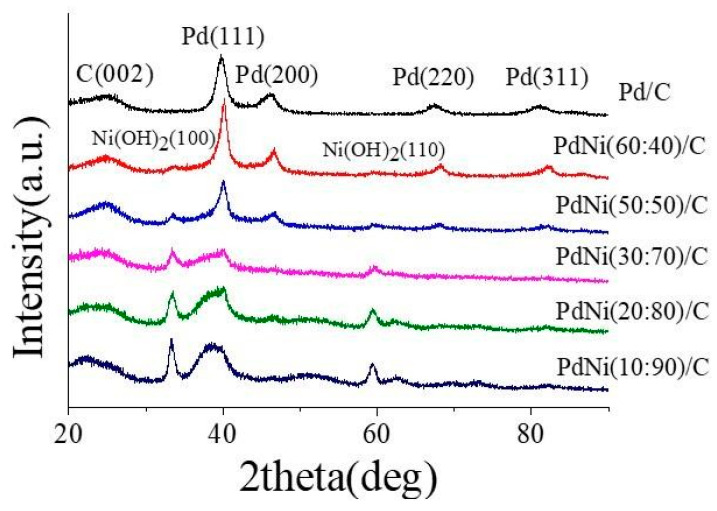
XRD pattern of Pd/C and different ratios of PdNi on carbon black.

The structural information of the PdNi/C electrocatalyst composite material was obtained through XRD, as shown in Figure 2. Additionally, Pd/C was prepared for comparison, confirming the presence of Pd and Ni(OH)_2_ particles. The carbon carrier exhibits a PdNi catalyst, and the broad peak at approximately 25° in 2θ can be attributed to the graphite (002) of Vulcan XC-72R carbon black. In the case of the carbon black loaded on PdNi, the diffraction peak was prominent, indicating a significant presence of graphite lattice in the carbon support. For the PdNi/C catalyst, four peaks near the 2θ values of 40.1°, 46.2°, 67.5°, and 82.1° were assigned to the Pd, with crystallographic planes corresponding to (111), (200), (220) and (311) of the face-centered cubic (FCC) crystallized Pd. On the other hand, the small peaks at the 2θ values of approximately 33.3° and 59.4° can be attributed to Ni(OH)_2_, corresponding to (100) and (110), respectively. The higher the weight percentage of Ni in the sample, the greater the intensity of these observed peaks. Additionally, in the presence of Ni, the diffraction peaks were found to shift to smaller angles compared to the Pd/C, indicating that some Ni has entered the Pd lattice, forming a PdNi alloy. At a higher Pd content, the Pd particles exhibited a stronger crystalline phase, while samples with a lower Pd content were in a more amorphous state, resulting in the absence of Pd(200) and Pd(311) peaks. Furthermore, as the Ni loading decreases, the peaks of Ni(OH)_2_ may become less pronounced, possibly due to the poor crystallinity of the Ni nanoparticles. It has been confirmed that PdNi has been attached to carbon black, resulting in the preparation of the PdNi/C catalyst. Using Scherrer’s equation and the diffraction angle of the maximum peak (111), it can be determined that the grain size of Pd in the PdNi/C catalyst is less than 4.0 nm.

#### 3.1.2. Electrochemical Characterizations of PdNi/C

**Figure 3:**
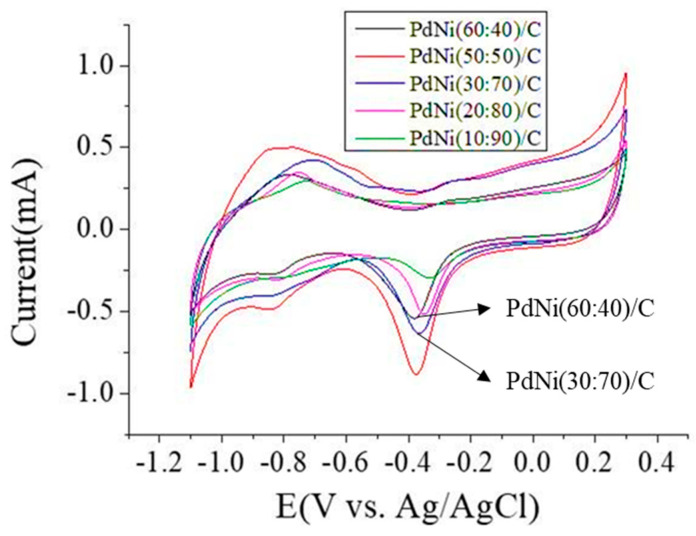
CV measurements of PdNi/C catalysts with various Pd and Ni wt.% in a 1 M KOH solution at a scan rate of 20 mVs^−1^.

The electrochemical behavior of PdNi/C was studied using cyclic voltammetry (CV). Different weight percentages of PdNi/C were tested in a 1 M KOH solution within a potential range of −1.1 V to 0.3 V. The working electrode was a glassy carbon coated with PdNi/C catalyst, the reference electrode was Ag/AgCl, and a Pt wire was used as the counter electrode. The electrochemical cyclic voltammograms obtained at a scan rate of 20 mV s^−1^ are shown in Figure 3. The CV curves are related to the activity of the electrode or composite material and the adsorption/desorption of hydroxide ions (OH^−^) on the Pd surface. The forward potential scan involves two processes, including the adsorption of hydrogen and the oxidation of Pd to PdO, with hydrogen adsorption occurring around −0.9 V to −0.4 V. Additionally, the PdO on the surface of the palladium is reduced back to palladium, occurring around −0.2 V to −0.6 V. This characteristic peak can be used to estimate the electrochemically active surface area (EASA) of the Pd catalyst. It is known that the catalytic activity of electrocatalysts depends on their electrochemically active surface area (EASA). Unlike platinum, samples based on palladium (Pd) make it difficult to evaluate the Coulomb charge of hydrogen monolayers on smooth palladium surfaces because Pd is essentially a hydrogen storage metal and inevitably absorbs hydrogen into the internal lattice space. Therefore, the adsorption/desorption peaks of hydrogen cannot be used to estimate the electrochemically active surface area (EASA). The EASA of PdNi/C is calculated based on the charge of the palladium oxidation-reduction peak. Equation (4) is shown as follows:

(4) $$\mathrm{EASA}=\frac{Q_{S}}{Q_{C}\times m}\times 10^{-4}$$

where *Qs* is the Coulomb charge (measured in μC) of the current peak reduced by PdO; *Qc* is the conversion factor and is typically considered to correspond to the reduction of PdO at 424 μC cm^−2^, where m is the loading amount of the Pd catalyst (mg) on the surface of the working electrode. The EASA calculation parameters are shown in Table 1, with the results indicating that the EASA of PdNi(50:50)/C reaches a maximum of 20.6 m^2^g^−1^, while the EASA of PdNi(10:90)/C is the lowest at 5.9 m^2^ g^−1^. At a lower Pd loading, the lack of Pd particle distribution on the support surface may be the reason for the smaller EASA. However, the decrease in EASA at 60 wt% Pd can be explained by the excessive coverage of Pd nanoparticles on the carbon support.

#### 3.1.3. X-Ray Diffraction Pattern of Ag/C

**Figure 4:**
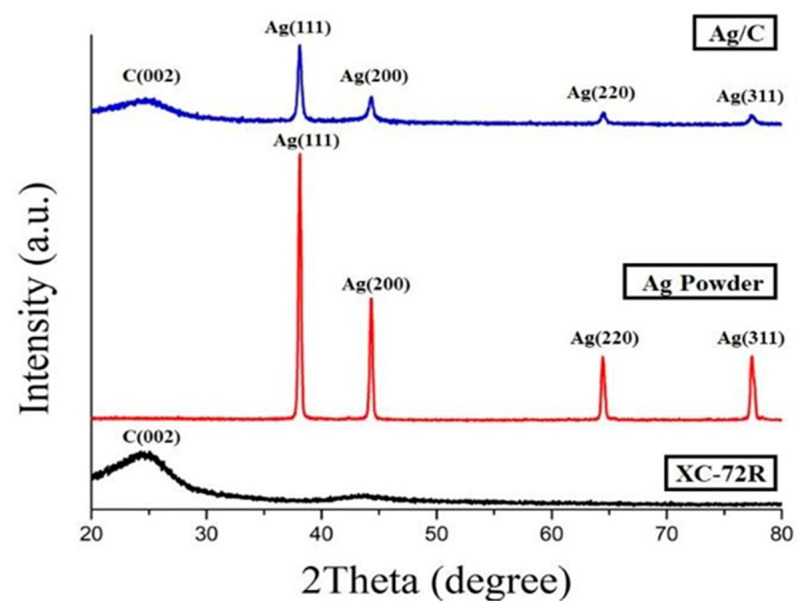
CV measurements of Ag powder, XC-72R carbon black and Ag/C.

Using the chemical reduction method to prepare an Ag/C catalyst, XRD analysis was conducted to determine whether the Ag nanoparticles were successfully attached to the carbon black support, as shown in Figure 4. The analysis indicated that carbon black XC-72R has a distinct peak at a 2θ value of 24.3 degrees, while the Ag nanoparticles showed distinct peaks at 2θ values of 38.1 degrees, 44.3 degrees, 66.4 degrees and 77.4 degrees. After comparison, it was found that the peak angles of the XC-72R and Ag particles are consistent, indicating that the lab-made Ag nanoparticles were successfully attached to XC-72R. Additionally, using Scherrer’s equation, the crystal size of Ag is calculated to be 25.71 nm.

#### 3.1.4. SEM Images of Ag/C

**Figure 5:**
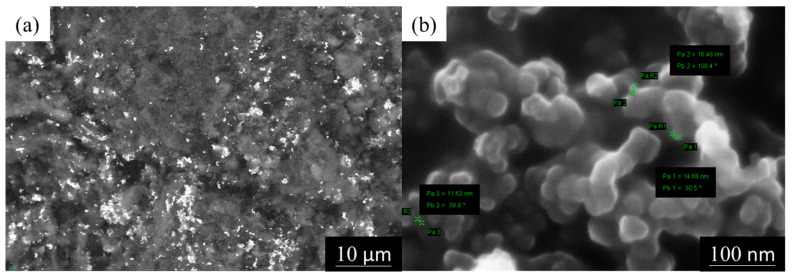
SEM images of Ag/C: (**a**) Ag nanoparticles are well distributed on XC-72R and (**b**) surface morphology of Ag nanoparticle catalysts.

It can be observed that the white image in Figure 5a represents the Ag nanoparticles, which are evenly dispersed on the carbon black support XC-72R. Subsequently, this was conducted using the SEM InLens mode set at a magnification of 150,000× to capture the Ag/C catalyst. Figure 5b shows the surface morphology of the Ag nanoparticle catalyst prepared by the chemical reduction method, with Ag’s nanoparticle sizes attached to the carbon black support ranging from 11 to 16 nm.

### 3.2. Anion-Exchange Membranes Analysis

#### 3.2.1. Ionic Conductivity of AEM

**Figure 6:**
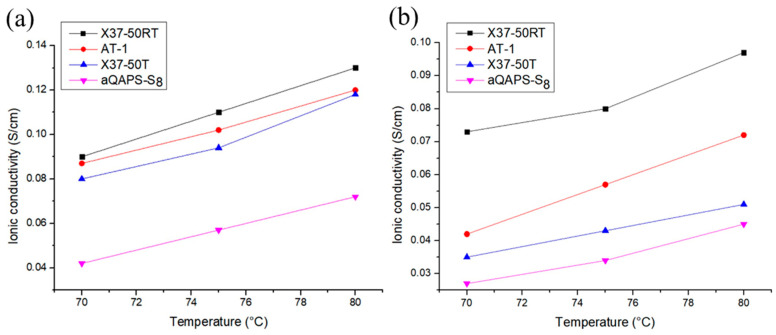
Ionic conductivity of membranes treated at various temperatures with (**a**) OH^−^ solutions and (**b**) Br^−^ solutions.

The parameters of the processed membranes are shown in Table 2 and Table 3. Subsequently, a four-point probe was used to measure the resistance, and after applying the formula, the ionic conductivity of the membranes was obtained. Figure 6 shows that the ionic conductivities of X37-50RT at 80 °C are 0.13 S/cm and 0.091 S/cm, resulting in the best performer among the four membranes. Additionally, experiments on power density also demonstrate a certain degree of correlation between high conductivity and higher power density.

#### 3.2.2. Water Uptake and Swelling Ratio of AEM

The water absorption of the membrane is calculated by the change in weight before and after wetting, as shown in the following Equation (5):

(5) $$Wut=\frac{W_{wet}-W_{dry}}{W_{dry}}\times 100\%$$

where *W _wet_* is the weight of the wet membrane, and *W_dry_* is the weight of the dry membrane. The weight of the dried membrane, measured at a size of 1 cm × 1 cm, was then immersed in deionized water at room temperature and allowed to equilibrate for 24 h. After 24 h, the membrane was quickly weighed after removing the surface water, and the data were recorded, as shown in Table 4. It showed that the water uptake of X37-50RT is the lowest among the four, and the change in membrane size is also relatively uniform, indicating that X37-50RT exhibits more stability when tested in fuel cells.

#### 3.2.3. Mechanical Properties of AEM

**Figure 7:**
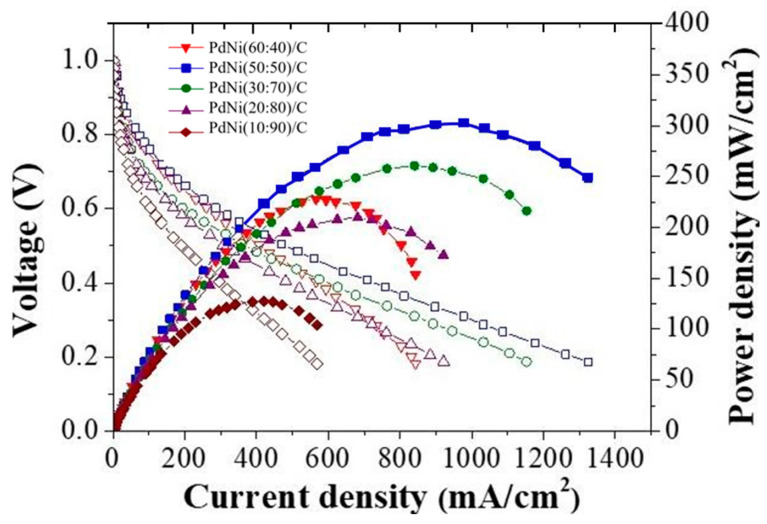
AEMFC performance of five different catalyst ratios of PdNi/C [39] with the use of AT-1 membrane. The hollow patterns and lines are I-V curves correlated to power density curves.

The mechanical properties comparison results of the dry and wet films are shown in Table 5 and Table 6. As a result, it can be observed that in the dry film state, AT-1 exhibits better properties in Young’s modulus, tensile strength and elongation at break. In contrast, X37-50RT becomes relatively brittle due to the requirement of first separating it in solution before drying it in the oven. However, in the wet film environment, X37-50RT shows the best mechanical properties, indicating that X37-50RT will have better durability when tested in the AEMFC. Figure 7 is a comparison of PdNi/C to Pt/C with the use of anion-exchange membrane AT-1 performance.

### 3.3. Single Fuel Cell Performance Test

#### 3.3.1. Performance of Anion-Exchange Membrane AT-1

The anode uses a PdNi/C catalyst with a loading capacity of 1.5 mg/cm^2^; the cathode uses a Pt/C catalyst with a loading capacity of 0.8 mg/cm^2^. As shown in Figure 7, The AEMFC power density of the PdNi(50:50)/C catalyst is the highest, which is consistent with the EASA calculation results. A higher Pd loading and less Ni loading in the PdNi(60:40)/C catalyst will result in more Pd coverage on the carbon region, possibly reducing the interactions between EASA and Pd–Ni, resulting in reduced AEMFC performance.

**Figure 8:**
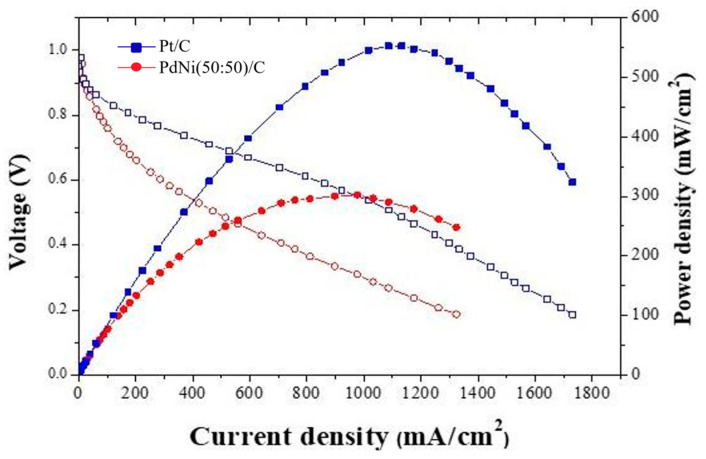
Power density curves of PdNi(50:50)/C (red line) and Pt/C (blue line) using AT-1 membrane [39].

The Pt/C and PdNi(50:50)/C is compared under the use of AT-1 membrane in order to know how well can it perform. Results show that Pt/C power density can reach around 550 mW/cm^2^, PdNi(50:50)/C power density can reach 302 mW/cm^2^. Although the power density of PdNi(50:50)/C is lower than Pt/C, it shows PdNi(50:50)/C can be alternative catalyst in AEMFC, as shown in Figure 8.

#### 3.3.2. Performance of aQAPS-S_8_ Membrane

**Figure 9:**
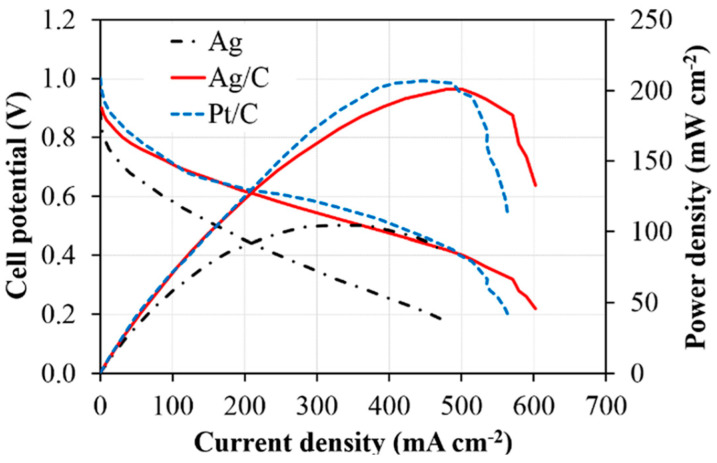
Power density curves of Ag, Ag/C and Pt/C using aQAPS-S_8_ membrane [40].

The Ag/C catalyst is used on the cathode, with a loading capacity of 1 mg/cm^2^. The anode uses a Pt/C catalyst with a loading capacity of 0.8 mg/cm^2^. It can be seen that the power density of the Ag/C catalyst is close to that of the Pt/C catalyst, which means that nano-grade silver can replace platinum as a catalyst for fuel cells, as shown in Figure 9.

#### 3.3.3. Performance of X37-50T Membrane

**Figure 10:**
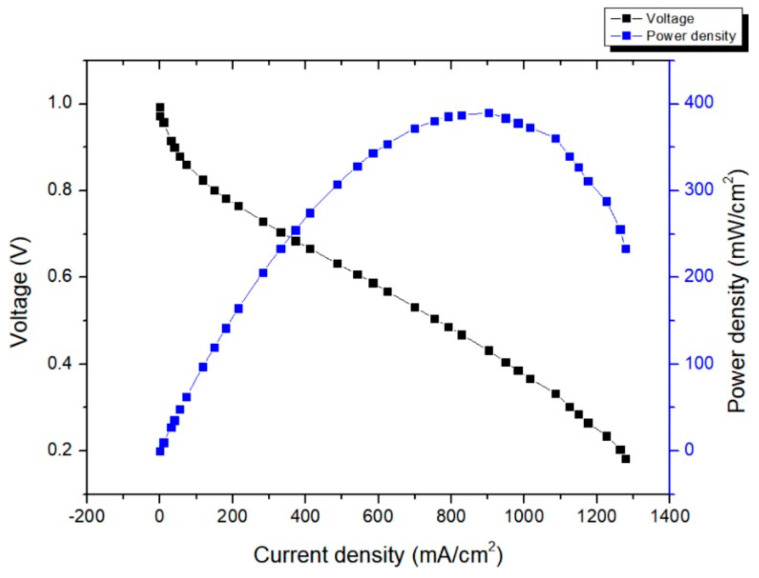
Power density curves of Ag, Ag/C and Pt/C using X37-50T membrane [40].

The Pt/C catalyst was used on both the anode and cathode; the loading amount was 0.8 mg/cm^2^, and the power density is shown in Figure 10. It is also found that the treated membrane may become curled and uneven, resulting in a lower power density compared to X37-50RT.

#### 3.3.4. Performance of X37-50RT Membrane

**Figure 11:**
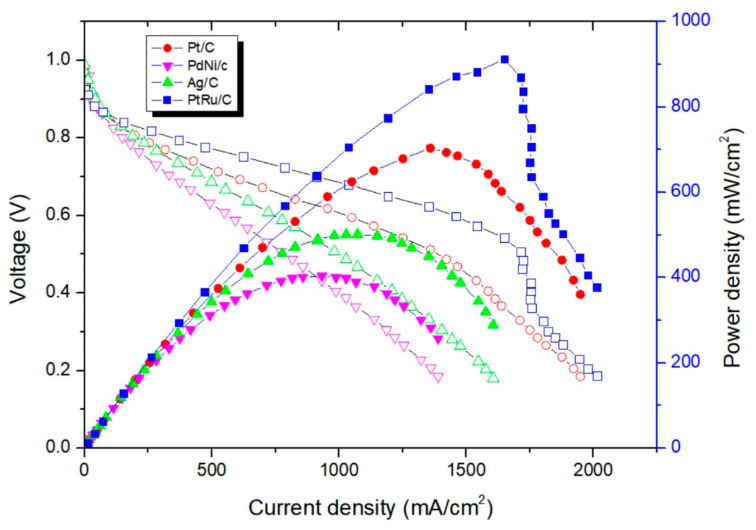
Power density curves of Pt/C, Ag/C, PdNi/C and PtRu/C catalysts using X37-50RT membrane.

As shown in Figure 11, the results show that the power density of using PdNi/C (50:50) as the anode and commercial Pt/C as the cathode is 400 mW/cm^2^, and the power density of using Ag/C as the cathode and commercial Pt/C as the anode is 510 mW/cm^2^. The power density of both the cathode and anode using commercial Pt/C is 703 mW/cm^2^; the anode using commercial PtRu/C and the cathode using commercial Pt/C have a power density of 912 mW/cm^2^. Compared with the other three membranes, the X37-50RT membrane efficiency of the PdNi/C, Ag/C, and Pt/C catalysts increased by 32%, 155% and 27%, respectively. Moreover, the X37-50RT membrane has the best power density performance.

#### 3.3.5. Comparison of Ionomers aQAPS-S_14_ to XB-7 Performance

**Figure 12:**
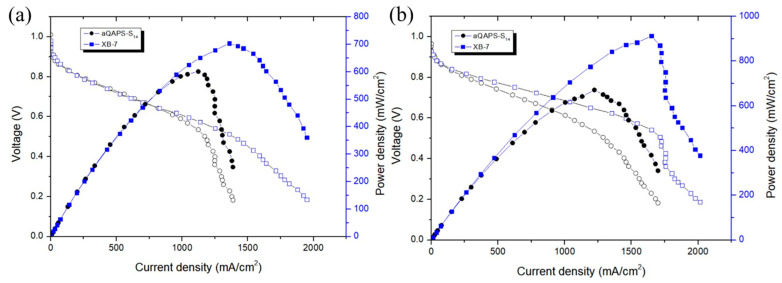
Power density curves of (**a**) using Pt/C catalysts and (**b**) using PtRu/C catalysts.

As shown in Figure 12, the Pt/C and PtRu/C catalysts were used, respectively, at a loading capacity of 0.8 mg/cm^2^. The 2 wt% aQAPS-S14 used dimethylformamide (DMF) as the solvent, while the 5 wt% XB-7 used ethanol as the solvent. Because ethanol contains hydrogen bonds and XB-7 has better coverage, its performance in power density and current density is better than that of 2 wt% aQAPS-S14.

## 4. Conclusions

This study successfully prepared PdNi/C catalysts with different weight percentages and Ag nanoparticle catalysts. The XRD analysis results show that Pd, Ni and Ag are successfully attached to carbon black. Using the Scherrer formula, the Pd crystal size in PdNi/C is estimated to be less than 4 nm, while the Ag crystal size in Ag/C is approximately in the range of a few tens of nanometers. Among the electrocatalysts prepared with different ratios of Pd and Ni, PdNi(50:50)/C exhibited the highest catalytic activity and good stability. This enhancement in electrocatalytic activity can be attributed to the higher electrochemically active surface area of PdNi(50:50)/C, making it a promising bifunctional electrocatalyst as an alternative to platinum. The results of the single-cell tests indicate that PdNi(50:50)/C exhibits the highest performance at 70 °C, with a peak power density of 302 mW/cm^2^, which is consistent with the CV results. Subsequently, different anion-exchange membranes and ionomers were tested, and the results showed that the XB-7 using ethanol solvent performed better than the aQAPS-S14 using dimethylformamide (DMF) solvent. This is attributed to the presence of hydrogen bonding in ethanol, which facilitates the mixing of the catalyst with IPA and DI water when preparing the catalyst solution. The X37-50RT membrane exhibited the most uniform size change after soaking, and its mechanical properties were better than those of the other three membranes. Additionally, its high ionic conductivity allows the AEMFC fuel cell to achieve a better power density and lifespan. The AEMFCs using the platinum catalyst and GDL-340 achieved a maximum power density of 703 mW/cm^2^ at 70 °C. The AEMFC using commercial PtRu/C at the anode and commercial Pt/C at the cathode demonstrated the highest peak power density of 912 mW/cm^2^. Non-platinum catalysts, such as palladium-nickel (PdNi) and nano-silver (Ag), can reach maximum power densities of 400 mW/cm^2^ and 510 mW/cm^2^ at 70 °C, showing improvements of 32%, 155% and 27% compared to other membranes. Although the power density is lower than the precious metal catalyst, the non-precious metal catalyst of anode electrode catalysts (PdNi/C) and cathode electrode catalysts (Ag/C) can be proven as substitutions of precious catalysts of Pt/C and PtRu/C. Therefore, the ion conductivity, water uptake and swelling ratio and mechanical characteristics can be referred to as selecting parameters for four AEMs for future AEMFC research.

## Figures and Tables

**Table 1:** Calculation of parameters involved in EASA using PdO reduction peak with PdNi/C catalyst.

Pd:Ni	Conversion Factor (μC cm^−2^)	Integral Coulomb (μC)	Metal Loading (mg)	EASA (m^2^ g^−1^)
60:40	424	5830	0.1	13.7
50:50	424	8740	0.1	20.6
30:70	424	6285	0.1	14.8
20:80	424	4400	0.1	10.4
10:90	424	2500	0.1	5.9

**Table 2:** Size chart of the four membranes after treatment with OH^-^ solution.

Parameters	aQAPS-S_8_	AT-1	X37-50RT	X37-50T
Thickness (μm)	35	35	83	68
Size (mm)	12.88	13.55	11.28	12.58
Electrode space (mm)	4.35	4.35	4.35	4.35

**Table 3:** Size chart of the four membranes after treatment with Br^-^ solution.

Parameters	aQAPS-S_8_	AT-1	X37-50RT	X37-50T
Thickness (μm)	40	77	65	58
Size (mm)	12.37	13.15	12.46	11.38
Electrode space (mm)	4.35	4.35	4.35	4.35

**Table 4:** Water uptake of membranes.

Parameters	aQAPS-S_8_	AT-1	X37-50RT	X37-50T
Water uptake (%)	36	46	31	68
Length stability (%)	1.04	0.21	5.82	12.58
Width stability (%)	8.23	6.32	7.33	2
Thickness stability (%)	19.6	15.9	4.57	3.5

**Table 5:** Mechanical properties of membranes under dry conditions.

Parameters	aQAPS-S_8_	AT-1	X37-50RT	X37-50T
Young’s modulus (MPa)	1154	2018	1113	1041
Elongation at break (%)	5.3	14.1	1.3	1.4
Tensile strength (MPa)	16	59	10	7

**Table 6:** Mechanical properties of membranes under wet conditions.

Parameters	aQAPS-S_8_	AT-1	X37-50RT	X37-50T
Young’s modulus (MPa)	278	218	529	323
Elongation at break (%)	4.1	3.2	4.8	12.5
Tensile strength (MPa)	4	1	5	2

## Data Availability

Data sharing is not applicable to this article.
